# Roles of *TNF*-α gene polymorphisms in the occurrence and progress of SARS-Cov infection: A case-control study

**DOI:** 10.1186/1471-2334-8-27

**Published:** 2008-02-29

**Authors:** Shixin Wang, Maoti Wei, Yi Han, Keju Zhang, Li He, Zhen Yang, Bing Su, Zhilun Zhang, Yilan Hu, Wuli Hui

**Affiliations:** 1 Proteomics Laboratory, Medical College of Chinese People's Armed Police Force, Tianjin, China; 2 Department of Epidemiology, Medical College of Chinese People's Armed Police Force, Tianjin, China; 3 Department of Respiratory, Pingjin Hospital, Medical College of Chinese People's Armed Police Force, Tianjin, China; 4 Department for Emergency Diseases, Center for Disease Control and Prevention, Tianjin, China

## Abstract

**Background:**

Host genetic factors may play a role in the occurrence and progress of SARS-Cov infection. This study was to investigate the relationship between tumor necrosis factor (TNF)-*α *gene polymorphisms with the occurrence of SARS-CoV infection and its role in prognosis of patients with lung interstitial fibrosis and femoral head osteonecrosis.

**Methods:**

The association between genetic polymorphisms of *TNF-α *gene and susceptibility to severe acute respiratory syndromes (SARS) was conducted in a hospital-based case-control study including 75 SARS patients, 41 health care workers and 92 healthy controls. Relationships of TNF-α gene polymorphisms with interstitial lung fibrosis and femoral head osteonecrosis were carried out in two case-case studies in discharged SARS patients. PCR sequencing based typing (PCR-SBT) method was used to determine the polymorphisms of *TNF-α *gene in locus of the promoter region and univariate logistic analysis was conducted in analyzing the collected data.

**Results:**

Compared to TT genotype, the CT genotype at the -204 locus was found associated with a protective effect on SARS with OR(95%*CI*) of 0.95(0.90–0.99). Also, TT genotype, CT and CC were found associated with a risk effect on femoral head necrosis with ORs(95%*CI*) of 5.33(1.39–20.45) and 5.67(2.74–11.71), respectively and the glucocorticoid adjusted OR of CT was 5.25(95%CI 1.18–23.46) and the combined (CT and CC) genotype OR was 6.0 (95%*CI *1.60–22.55) at -1031 site of *TNF-α *gene. At the same time, the -863 AC genotype was manifested as another risk effect associated with femoral head necrosis with OR(95%*CI*) of 6.42(1.53–26.88) and the adjusted OR was 8.40(95%CI 1.76–40.02) in cured SARS patients compared to CC genotype.

**Conclusion:**

SNPs of *TNF-α *gene of promoter region may not associate with SARS-CoV infection. And these SNPs may not affect interstitial lung fibrosis in cured SARS patients. However, the -1031CT/CC and -863 AC genotypes may be risk factors of femoral head necrosis in discharged SARS patients.

## Background

*TNF*, the gene encoding tumour necrosis factor (TNF), resides in the central part (class III region) of the major histocompatibility complex (MHC) surrounded by a large number of other immunological genes [1]. Because of the special locus of this gene, it can be deduced that this gene may associate with many diseases, and this hypothesis was confirmed by many research results [2,3]. TNF-α is a key mediator of the inflammatory response and is critical for host defense against a wide variety of pathogenic microbes. However, the over-expression of this cytokine may lead to badness in disease recovery. The dual role of TNF, acting as an agent of both innate immunity and inflammatory pathology, poses a considerable challenge for gene regulation [2], and this regulation mainly located on promoter region of this gene. The capacity for cytokine production in an individual has a major genetic component, and striking differences existed among individuals in terms of their ability to produce cytokines. Several biallelic polymorphisms had been described within the *TNF-α *gene, including seven in the promoter region at positions -1031T→C, -863C→A, -857C→T, -376G→A, -308G→A, -238G→A and -163G→A base pairs from the transcription start site [4,5]. Moreover, a number of studies had shown that the *TNF-α *promoter polymorphism had a significant effect on its transcriptional activity [6,7].

Severe acute respiratory syndrome (SARS) is a newly described human infectious disease caused by a novel coronavirus-SARS-CoV. SARS-CoV infection is important because of its high infectivity and unpredictable clinical course, which is characterized by a high mortality rate [8]. Till now, many researchers had reported that susceptibilities to infection SARS-CoV may associated with *HLA*, *MXA, OAS-1 *and *CLEC4M *gene polymorphisms [9-13], yet these results were variable in different populations. For example, Ng reported that SARS-CoV infection was associated with HLA-B*0703 and HLA-DRB1*0301 in HongKong population [9], however, Lin's results showed that HLA-B*4601 and HLA-B*5401 were closely related to SARS-CoV infection [10]. Chan reported that *CLEC4M *was attributed to SARS-CoV infection [13], but Zhi's results failed to support this conclusion.^7 ^These differences may be attributed to the study population used in each report, also the complex mechanism infection to SARS-CoV should be considered as another factor of these differences. In order to explore more host factors influencing the occurrence of SARS-CoV infection, we studied the polymorphisms of *TNF-α *gene at the promoter region, which had been ascribed to polymorphisms within the regulatory regions or signal sequences of cytokine genes [14].

After discharging from hospital, interstitial lung fibrosis was observed in SARS patients. Clinical data showed that the prevalence rate of this change was 21%(42/200) in cured SARS patients nine months from the discharge [15]. TNF-α was one of the earliest cytokines implicated in the pathogenesis of lung fibrosis diseases and, together with IL-1, has been found to over-expressed in regenerated type II pneumocytes in human lung, thus enhanced fibroblast proliferation [16]*TNF-α *polymorphisms have been discovered significantly associated with increased risk of developing pulmonary fibrosis [17,18]. Given that genetic variation may potentially alter inflammation and fibrosis in the lung, the aim of this case-case control study was to examine the *TNF-α *polymorphisms with interstitial lung fibrosis in SARS patients.

In spite of interstitial lung fibrosis in cured SARS patients, another sequela – femoral head necrosis was also observed in this population and the prevalence rate was 22.07%(49/221) and 23.1%(18/78) in Tianjin and Beijing patients respectively [15,19]. The cause of this disease was still unknown and there were arguments about it. For example, some author considered SARS-CoV as the cause of femoral head necrosis, yet other authors disagreed with this view [20,21]. Previous studies showed that femoral head necrosis may caused by hormone usage [20], yet our data failed to agree with this point. So, it need further study to explore the cause of this sequela and *TNF-α *polymorphisms were considered first in this report.

In this paper, we aimed to study whether polymorphisms in *TNF*-*α *promoter region were associated with SARS-CoV infection, development, and progression of interstitial lung fibrosis and femoral head necrosis in cure SARS patients.

## Methods

### Subjects

This study was reviewed and approved by ethics committees in the Medical College of CPAFP. The study population comprised 75 SARS patients in Pingjin hospital, Tianjin, China, 41 health care workers of the same hospital, who had come into contact with SARS patients but had not developed into SARS, and 66 individuals having no contact history with SARS patients. Among 75 SARS patients, 55 could be classified into severe and light SARS according to their clinical condition history during the hospitalized period and this population also had the history of hormone therapy by reviewing the clinical treatment. Anti-SARS-CoV antibodies of the serum samples were tested by SARS ELISA kits (Huada Diagnostics Ltd, Beijing, China).

Considered that the progression of interstitial lung fibrosis or femoral head necrosis may be affected by hormone therapy, hormone using dosage, method and lasting period were considered in this study when analyzing the associations between gene polymorphisms with disease. Three kinds of hormone were used in SARS patients including methylprednisolone, deltadehydrocortisone and dexamethasone. In order to simplify analyzing, deltadehydrocortisone and dexamethasone dosage were calculated into methylprednisolone using the following equation: 4 mg methylprednisolone = 5 mg deltadehydrocortisone = 0.75 mg dexamethasone. Lash therapy means more than 320 mg methylprednisolone were used in a single day.

Cured SARS patients with interstitial lung fibrosis were diagnosed by respiratory experts according to CT results following the standard proposal for therapy and diagnosis of SARS patients issued by Chinese Ministry of Health in 2004 [22]. Interstitial lung fibrosis of SARS patients manifested as irregular patch and strip shadow or high density strip shadow and honeycomb interstitial lung fibrosis, these changes could combine with the bronchiectasis.

Femoral head necrosis was diagnosed using magnetic resonance imaging (MRI). An MRI scan of a normal femoral head would show uniformly high signal intensity on T1- and T2-weighting throughout the femoral head. Agree with one of the following image could be diagnosed as femoral head necrosis in SARS patients: Abnormal signal with clear margin in cartilage of femoral head, or double thread image, or fracture or joint dent under cartilage, or T1WI low signal, T2WI and STIR high signal of the marrow cavity edema with blur edge [22].

### Genomic DNA extraction and polymerase chain reaction

Leucocytes were isolated within 12 h of blood collection using Percoll reagent. Then genomic DNA was extracted using cell DNA extraction kit (Tiangen BioTec Co, Beijing, China, patch number: 2004-08-13) according to the manufacturer's instructions. Primers were designed according to Gewaltig [23]. Standard 50-μL polymerase chain reactions (PCRs) contained 5 μl(6.7 μM) forward primer 5'-GATGGACTCACCAGGTGAG-3', 5 μl(6.7 μM) reverse primer 5'-CTCATGGTGTCCTTTCCAGG-3', 5 μl buffer [150 mm (NH_4_)_2_SO_4_, 500 mM Tris-Cl(pH = 8.8), 500 μM EDTA-Na_2_,15 mM MgCl_2_,100 mM β-Mercaptoethanol], 0.5 μL DNA polymerase (Tiangen BioTec Co, Beijing, China), 3 μl DNA template. Amplification was carried out in a thermal cycler TC312 (Techne, Duxford Cambridge, UK) with cycle parameters of 5 min at 94°C (initial denaturation), 35 rounds of 94°C 30 s, 60°C 100 s and 72°C 150 s, and a final extension for 5 min at 72°C. The reactions were carried out in molecular BioProducts 200 μL capped tubes, as these gave optimal heat transfer in the thermal cycler.

### Sequencing of *TNF-α *gene fragments

The *TNF*-α gene 1279 bp fragments in this paper were sequenced in double directions with forward primer 5'-GATGGACTCACCAGGTGAG-3' and reverse primer 5'-CTCATGGTGTCCTTTCCAGG-3' and Invitrogen company (Invitrogen Co, Shanghai, China) using ABI 373 thermal cycler carried out this job. The homozygote genotype of each SNP site manifested as a single peak, yet the heterozygote with an ambiguous nucleotide position of a double color peak in the Big Dye chemistry pictures. According to reading the sequence graphs, the genotype was determined.

### Statistics

The differences in values between two groups were evaluated by Chi analysis for frequencies or student *t *test for quantitative index and binary logistic regression was done using SPSS 11.5 software (SPSS Inc, Chicago, Illinois, USA).

## Results

### Demographic characteristics of the populations

A total of 75 SARS patients, 41 health care workers and 66 individuals were included in this study. All the populations were Chinese Han ethnic. The mean age was 35.0 years for SARS, 35.7 for health care workers and 30.1 for individual controls (SARS *Vs *HCW, *P *> 0.05; SARS *Vs *individual control, *P *> 0.05). The proportion of male was 30.6% in SARS, 26.8% in health care workers and 69.5% in individual controls (SARS *Vs *HCW, *P *> 0.05; SARS *Vs *individual control, *P *< 0.05). The sera positive rate anti-SARS-CoV antibody was 100.0% in SARS, significantly higher than that of health care workers and individual controls (SARS *Vs *HCW, *P *< 0.05; SARS *Vs *individual control, *P *< 0.05) (Table 1).

**Table 1:** Demographic characteristics of the populations

Characteristics	SARS (N = 75)	Health care workers (N = 41)	Control (N = 92)
Age(years), mean [range]	35.0 [16-78]	35.7 [25-55]	30.1 [17-56]
Gender (Male/Female)	23/52	11/30	68/24
Positive rate of anti-SARS-CoV Ab	100.0%(75/75)	2.4%(1/41)	0.0%(0/92)

### *TNF-α *polymorphisms and SARS-Cov infection

*TNF-α *genotype frequencies were variable in SARS, health care workers and individual controls. There were no differences of *TNF-α *genotype distribution at the -1031(T→C), -863(C→A), -572(A→C), -308(G→A) and -238(G→A) among the three populations. However, the CT genotype was less frequent in SARS group when compared with individual controls at the -204 locus (*X*^2 ^= 4.20, *P *= 0.04). Compared to TT genotype, the CT genotype at the -204 locus were found associated with a protective effect on SARS with OR(95%*CI*) of 0.95(0.90–0.99) (Table 2).

**Table 2:** *TNF*-α gene polymorphism in promoter region in cured SARS patients, health care workers and individual control

SNP site	SARS (%)	Health Care Workers (%)	Individual Control (%)	OR^1 ^(95%CI)	OR^2 ^(95%CI)
-1031					
Genotype					
TT	45(63.4)	25(64.1)	61(67.8)	-	-
CT	23(32.4)	13(33.3)	27(30.0)	0.98(0.43–2.27)	1.12(0.59–2.27)
CC	3(4.2)	1(2.6)	2 (2.2)	1.67(0.17–16.88)	2.03(0.33–12.68)
Allele					
T	79.6	80.8	74.5		
C	20.4	19.2	25.5		
-863					
Genotype					
CC	55(73.3)	29(72.5)	64(70.3)	-	-
AC	19(25.3)	11(27.5)	27(29.7)	0.91(0.38–2.17)	0.82(0.41–1.63)
AA	1(1.3)	0	0	0.98(0.94–1.07)^&^	2.16(1.78–2.63)^&^
Allele					
C	86.0	86.3	85.2		
A	14.0	13.7	14.8		
-857					
Genotype					
CC	56(74.7)	31(77.5)	66(72.5)	-	-
CT	19(25.3)	8(20.0)	25(27.5)	1.32(0.52–3.35)	0.90(0.45–1.79)
TT	0	1(2.5)	0	1.03(0.97–1.1)^$^	-
Allele					
C	131(87.3)	70(87.5)	157(86.3)		
T	19(12.7)	10(12.5)	25(13.7)		
-572					
Genotype					
AA	73(97.3)	40(97.6)	90(97.8)	-	-
AC	2(2.7)	1(2.4)	2(2.2)	1.10(0.10–12.46)	1.23(0.17–8.97)
Allele					
A	98.7	98.8	98.9		
C	1.3	1.2	1.1		
-308					
Genotype					
GG	67(89.3)	39(95.1)	88(95.7)	-	-
AG	7(9.1)	2(4.9)	4(4.3)	2.04(0.40–10.30)	2.30(0.65–8.18)
AA	1(1.3)	0	0	0.98(0.96–1.01)^&^	2.31(1.93–2.77)^&^
Allele					
G	94.0	97.6	100.0		
A	6.0	2.4	0.0		
-238					
Genotype					
GG	65(86.7)	39(95.1)	84(91.3)	-	-
AG	10(13.3)	2(4.9)	8(8.7)	3.00(0.63–14.41)	1.62(0.60–4.32)
Allele					
G	93.3	97.6	95.7		
A	6.7	2.4	4.3		
-204					
Genotype					
TT	75(100)	41(100.0)	87(94.6)	-	-
CT	0(0.0)	0	5(5.4)	-	0.95(0.90–0.99)^^$^^
Allele					
T	100.0	100.0	97.3		
C	0.0	0.0	2.7		
-163					
Genotype					
GG	74(98.7)	41(100.0)	87(94.6)	-	-
CG	1(1.3)	0	5 (5.4)	0.98(0.96–1.01)^&^	0.24(0.03–2.06)
Allele					
G	99.3	100.0	97.3		
C	0.7	0.0	2.7		

Note: OR^1 ^was calculated by comparing SARS with HCW;

OR^2 ^was calculated by comparing SARS with individual control;

&: Odds ratio replaced with SARS group; $: Odds ratio replaced with control

According to the clinical history, symptoms of SARS patients were classified into light and severe. Because of the complicated clinical condition during SARS outbreak, some patients' histories were incomplete and could not be classified following the severity standard [22]. The severe SARS referred to those with one or more of the following: (1) dyspnea, more than 30 times per min respiratory frequencies in still condition;(2) oxygenation index less than 300 mmHg; (3) shock or multiple organ dysfunction syndrome. Among all 75 patients, fifty-four were classified into light and severe. And there were no association of *TNF-α *polymorphisms and SARS severity (Table 3).

**Table 3:** *TNF*-α gene polymorphism and the progress of SARS

SNP site	Severe SARS(%)	Light SARS(%)	OR* (95%CI)	*P*
-1031				
Genotype				
TT	13(61.9)	22(73.3)		
CT	7(33.3)	8(26.7)	1.48(0.44–5.04)	0.53
CC	1(4.8)	0(0.0)	2.69(1.75–4.14)^^&^^	0.39
Allele				
T	78.6	86.7		
C	21.4	13.3		
-863				
Genotype				
CC	19(79.2)	24(80.0)		
AC	5(20.8)	6(20.0)	1.05(0.28–3.98)	0.60
Allele				
C	89.6	90.0		
A	10.4	10.0		
-857				
Genotype				
CC	15(62.5)	24(80.0)		
CT	9(37.5)	6(20.0)	2.40(0.71–8.11)	0.15
Allele				
C	39(81.2)	54(90.0)		
T	9(18.8)	6(10.0)		
-572				
Genotype				
AA	24(100.0)	29(96.7)		
AC	0(0.0)	1(3.3)	1.83(1.43–2.34)	1.00
Allele				
A	100.0	98.3		
C	0.0	1.7		
-308				
Genotype				
GG	20(83.3)	27(90.0)		
AG	4(16.7)	2(6.7)	2.70(0.45–16.22)	0.39
AA	0(0.0)	1(3.3)	1.74(1.36–2.23)^^$^^	1.00
Allele				
G	100.0	93.3		
A	0.0	6.7		
-238				
Genotype				
GG	20(83.3)	27(90.0)		
AG	4(16.7)	3(10.0)	1.80(0.36–8.96)	0.69
Allele				
G	91.70	95.0		
A	8.3	5.0		
-204				
Genotype				
TT	24(100.0)	30(100.0)		
Allele				
T	100.0	100.0		
-163				
Genotype				
GG	24(100.0)	30(100.0)		
Allele				
G	100.0	100.0		

Note:*: OR was calculated using light SARS as control.

&: Odds ratio replaced with severity SARS group;

$: Odds ratio replaced with light SARS

### Glucocorticoid using in SARS with interstitial lung fibrosis or femoral head necrosis

Glucocorticoid using dosage, method and lasting period were not associated with interstitial lung fibrosis or femoral head necrosis in binary logistic analysis in SARS patients (Table 4). And there was no difference of hormone using dosage between the interstitial lung fibrosis and non-interstitial lung fibrosis group(*t *= 0.72, *P *= 0.47) and this trend was also observed in the femoral head necrosis and non-femoral head necrosis group(*t *= 1.90, *P *= 0.064) (Table 5).

**Table 4:** Binary logistic analysis of hormone using in SARS patients

Factors	Interstitial lung fibrosis				Femoral head necrosis			
	
	*P*	OR	95.0% C.I.		*P*	OR	95.0% C.I.	
Glucocorticoid	0.97	1.00	0.99	1.00	0.11	1.00	0.99	1.00
Time	0.78	0.97	0.81	1.18	0.96	1.00	0.84	1.18
Lash therapy	0.51	2.13	0.23	20.13	0.27	0.32	0.04	2.43
Constant	0.68	3.48			0.13	88.28		

**Table 5:** Cumulative hormone usage (mg) in cured SARS patients classified according to interstitial lung fibrosis or femoral head necrosis

	Mean ± SD	Median	*P*_25_	*P*_75_
Interstitial lung fibrosis^&^				
Yes	5188.13 ± 2037.56	5140.00	3110.00	7288.75
No	4493.11 ± 2528.91	3895.00	2720.00	5975.00
Femoral head necrosis^&&^				
Yes	5672.31 ± 2328.61	6090.00	3437.50	7872.50
No	4187.81 ± 2389.71	3807.50	2355.00	5707.50

&: hormone dosage between interstitial lung fibrosis and non-interstitial lung fibrosis, *t *= 0.72, *P *= 0.47;

&&: hormone dosage between femoral head necrosis and non-femoral head necrosis, *t *= 1.90, *P *= 0.064

### *TNF-α *polymorphisms and interstitial lung fibrosis

Allele frequencies of *TNF-α *polymorphisms were listed in Table 6 and there were no significant differences between interstitial lung fibrosis and non-interstitial lung fibrosis in SARS patients at promoter region of *TNF-α *gene.

**Table 6:** *TNF*-α gene polymorphisms and the occurrence of interstitial lung fibrosis in SARS patients

SNP site	Interstitial lung fibrosis(%)	Non-interstitial lung fibrosis(%)	OR* (95%C.I.)	*P*
-1031				
Genotype				
TT	14(70)	12(57.1)		
CT	6(30)	8(38.1)	0.64(0.17–2.38)	0.51
CC	0	1(4.8)	1.08(0.93–1.27)^‡^	0.48
Allele				
T	85.00	76.19		
C	15.00	23.81		
-863				
Genotype				
CC	17(77.3)	16(72.7)		
AC	5(22.7)	6(27.3)	0.78(0.20–3.08)	0.73
Allele				
C	88.64	38		
A	11.36	13.64		
-857				
Genotype				
CC	15(68.8)	17(77.3)		
CT	7(31.8)	5(22.7)	1.59(0.42–6.07)	0.50
Allele				
C	84.09	88.64		
T	15.91	11.36		
-572				
Genotype				
AA	21(95.5)	22(100)		
AC	1(4.5)	0	0.96(0.87–1.05)^&^	0.50
Allele				
A	97.73	100.00		
C	2.27	0.00		
-308				
Genotype				
GG	21(95.5)	18(81.8)		
AG	1(4.5)	4(18.2)	0.21(0.02–2.10)	0.17
Allele				
G	97.73	90.9		
A	2.27	9.1		
-238				
Genotype				
GG	20(90.9)	18(81.8)		
AG	2(9.1)	4(18.2)	0.45(0.07–2.76)	0.33
Allele				
G	95.45	90.9		
A	4.55	9.1		

Note:*:OR was calculated using non-interstitial lung fibrosis as control.

&: Odds ratio replaced with interstitial lung fibrosis group;

‡: Odds ratio replaced with non-interstitial lung fibrosis group

### *TNF*-α polymorphism and femoral head necrosis

Allele frequencies of *TNF*-α gene were compared in SARS patients between femoral head necrosis and non-femoral head necrosis (Table 7). The -1031 CT and CC genotypes were more frequent in SARS patients with femoral head necrosis(53.7% and 6.7%, respectively) than in non-femoral head necrosis(20.0% and 0.0%, respectively). Compared to TT genotype, CT and CC were found associated with a risk effect on femoral head necrosis with ORs(95%*CI*) of 5.33(1.39–20.45) and 5.67(2.74–11.71), respectively. The adjusted OR of CT was 5.25(95%CI 1.18–23.46) and the combined (CT and CC) genotype OR was 6.0 (95%CI 1.60–22.55). Also, the -863 AC genotype accounted for 43.7% of femoral head necrosis group but 10.8% of non-femoral head necrosis. Compared to CC genotype, the AC genotype was manifested as another risk effect associated with femoral head necrosis with OR(95%CI) of 6.42(1.53–26.88) and the adjusted OR was 8.40(95%CI 1.76–40.02) in cured SARS patients.

**Table 7:** *TNF*-α gene polymorphism and femoral head necrosis in cured SARS patients.

SNP site	Femoral head necrosis (%)	Non-femoral head necrosis (%)	OR* (95%C.I.)	*P*
-1031				
Genotype				
TT	6(40)	28(80)		
CT	8(53.7)	7(20)	5.33(1.39–20.45)	0.01
CC	1(6.7)	0	5.67(2.74–11.71)^$^	0.04
Allele				
T	66.67	90.00		
C	33.33	10.00		
-863				
Genotype				
CC	9(56.3)	33(89.2)		
AC	7(43.7)	4(10.8)	6.42(1.53–26.88)	0.01
Allele				
C	78.12	94.59		
A	21.88	5.41		
-857				
Genotype				
CC	12(75)	26(70.3)		
CT	4(25)	11(29.7)	0.78(0.21–2.99)	0.73
Allele				
C	87.50	85.14		
T	12.50	14.86		
-572				
Genotype				
AA	16(100)	36(97.3)		
AC	0	1(2.7)	1.44(1.21–1.73)^‡^	0.70
Allele				
A	100	98.7		
C	0	1.3		
-308				
Genotype				
GG	14(87.5)	32(86.5)		
AG	2(12.5)	4(10.8)	1.14(0.19–6.98)	0.89
AA	0	1(2.7)	1.44(1.19–1.74)^‡^	0.70
Allele				
G	93.37	91.89		
A	6.25	8.11		
-238				
Genotype				
GG	13(81.3)	33(89.2)		
AG	3(18.7)	4(10.8)	1.90(0.37–9.71)	0.35
Allele				
G	90.62	94.59		
A	9.38	5.41		

Note: *:OR was calculated using non-femoral head necrosis as control.

$:OR replaced by femoral head necrosis;

‡: OR estimated by non-femoral head necrosis;

## Discussion

Four years after SARS occurrence, many problems still remained unknown to us. Till now, many researchers have reported that susceptibility to infection SARS-CoV may associate with HLA, MXA, OAS-1 and CLEC4M gene polymorphisms, yet the results are variable in different populations [9-13]. These differences may be attributed to the study population used in each report, also the complex mechanism infection to SARS-CoV should be considered as another factor of these differences. In order to explore more host factor influence the occurrence of SARS-CoV infection, we studied the polymorphisms of *TNF-α *gene at the promoter region, which have been ascribed to polymorphisms within the regulatory regions or signal sequences of cytokine genes [14]. Allele distributions at -1031, -863, -857, -572, -238 and -163 were almost the same among the SARS, the health care workers and individual controls, but a higher A allele frequency in SARS population when compared with the control at the -308 locus(*X*^2 ^= 8.96, *P *= 0.003). Though previous study showed that *TNF-α *-308 AG genotype was associated with the clearance of Hepatitis B virus and the infection of *Helicobacter pylori *cagA subtype infection [24,25], our results failed to show the role of this locus in SARS-CoV infection and this conclusion agreed with that of Chong WP *et al *[26]. We found that there was a weak protective effect of CT genotype at -204 locus of *TNF-α *gene against SARS-CoV infection. The -204 locus was a new discovered SNP site of *TNF-α *promoter region and its role in infectious diseases might need further study. At the same time, no obvious association between the polymorphisms of *TNF-α *promoter region with the severity of SARS was observed. However, Lu reported that the -238G/A polymorphism of *TNF-α *associated with the outcomes of hepatitis B virus infection [27]. Thus, the roles of *TNF-α *gene in infectious diseases should be further studied.

TNF-α, a key mediator of the inflammatory response, is critical for host defense against a wide variety of pathogenic microbes, but a higher concentration of this cytokine may cause severe pathology. The capacity for cytokine production in an individual has a major genetic component, and striking differences existed among individuals in terms of their ability to produce cytokines. A number of studies had shown that the *TNF-α *promoter polymorphism has a significant effect on transcriptional activity [6,7]. During the process of SARS-CoV infection, there was a cytokine storm in patients including IL-1, IL-2, IL-4, IL-6, IL-8, IL-10, IFN-γ, TNF-α and TGF-β_1 _[28]. The elevated levels of pro-inflammatory cytokines which may cause immuno-mediated damage to lung and other organs, resulting in acute lung injury and, subsequently, multi-organ dysfunction [29]. So, *TNF-α *genetic variation may potentially alter inflammation and fibrosis in the lung. After discharging from hospital, interstitial lung fibrosis was observed in cured SARS patients and the prevalence rate was 21%(42/200). TNF-α was one of the earliest cytokines implicated in the pathogenesis of lung fibrosis disease and, together with IL-1, has been found to over-express in regenerating type II pneumocytes in human lung, thus enhancing fibroblast proliferation [16].*TNF-α *genetic polymorphisms have been found significantly associated with increased risk of developing pulmonary fibrosis [17,18]. During the progress of idiopathic pulmonary fibrosis, activated epithelial cells are thought to release potent fibrogenic molecules and cytokines, such as TNF-α and TGF-β_1_, which in turn foster the transformation of fibroblasts into myofibroblasts and promote their production of extracellular matrix molecules and a vicious cycle of injury and abnormal epithelial healing sets the stage for progressive fibrosis and architectural distortion of the lung parenchyma [7]. However, our data failed to show that alleles at -1031(T→C), -863(C→A), -857(C→T), -572(A→C), -308(G→A) and -238(G→A) were related to interstitial lung fibrosis when compared with non-interstitial lung fibrosis in SARS patients. At the same time, -204 and -163 were homozygote genotype of TT and GG respectively in SARS patients and no relationship of genotype with interstitial lung fibrosis could be calculated. This result implicated that there maybe a different mechanism of interstitial lung fibrosis of SARS compared with idiopathic pulmonary fibrosis.

Femoral head necrosis, another sequela of discharged SARS patients, prevailed with a rate of 22.07%(49/221) in Tianjin [15]. However, the cause of this sequela was still unknown and there were arguments about it. For example, some author considered SARS-CoV as the cause of femoral head necrosis, yet other authors disagree with this view [20,21]. Previous studies showed that femoral head necrosis may caused by hormone usage, our data was far to agree with this conclusion. There was no obvious association between hormone using including hormone dosage, method and lasting period with femoral head necrosis in binary logistic analysis in SARS patients. We found that the -1031 CT and CC genotypes were more frequent in SARS patients with femoral head necrosis(53.7% and 6.7%, respectively) than in non-femoral head necrosis(20.0% and 0.0%, respectively). And CT and CC were related with a risk effect on femoral head necrosis with ORs (95%*CI*) of 5.33(1.39–20.45) and 5.67(2.74–11.71), respectively when compared to TT genotype. The hormone using adjusted OR of CT was 5.25(95%CI 1.18–23.46) and the combined (CT and CC) genotype OR was 6.0 (95%CI 1.60–22.55). Also, the -863 AC genotype accounted for 43.7% of femoral head necrosis group but 10.8% of non-femoral head necrosis. Compared to CC genotype, the AC genotype was manifested as another risk effect associated with femoral head necrosis with OR(95%CI) of 6.42(1.53–26.88) and the adjusted OR was 8.40(95%CI 1.76–40.02) in cured SARS patients. TNF-α can activate activity of osteoclasts and accelerate absorption of the bone and cartilage and induce occurrence of oxygen free radicals an d lipid peroxidation, which can induce ischemic necrosis of the femoral head [30]. So, the ploymophism *TNF-α *gene may be directly or indirectly attributed to the occurrence of femoral head necrosis in SARS patients.

## Conclusion

In conclusion, there may be no association of *TNF*-α polymorphisms in promoter region with SARS-Cov infection. Also, *TNF-α *gene polymorphisms may no affect the occurrence of interstitial lung fibrosis in cured SARS patients. However, the polymorphisms may relate with femoral head necrosis.

## Competing interests

The author(s) declare that they have no competing interests.

## Authors' contributions

SXW conceived of the study, and participated in its design and coordination.

MTW, co-first author, carried out the molecular genetic studies and drafted the manuscript.

YH, KJZ and LH carried out the molecular genetic studies

ZY, BS and ZLZ participated in field investigation and samples collection of the study

YLH and WLH participated in the design of the study and performed the statistical analysis

All authors contributed to writing of the final manuscript

All authors read and approved the final manuscript

## Pre-publication history

The pre-publication history for this paper can be accessed here:


